# Novel method for prediction of combinatorial phase-variable gene expression states

**DOI:** 10.1016/j.mex.2023.102392

**Published:** 2023-09-22

**Authors:** Jonathan Holmes, Lickson Munjoma, Christopher D. Bayliss

**Affiliations:** Department of Genetics and Genome Biology, University of Leicester, United Kingdom

**Keywords:** Phase variation, GeneScan, Campylobacter jejuni, Neisseria meningitidis, Simple sequence repeats, Microsatellites, Phasotype, Sweep-Corrected Phasotype Analysis

## Abstract

Short sequence repeat mediated phase variation results in diverse phenotype presentation in many bacteria including *Campylobacter* and *Neisseria* species. Current methods for identifying the expression states of phase-variable genes involve taking a high number of single colonies. This approach is subject to bias, sampling effects and high workloads that reduce the ability to perform intermediary sampling. The use of high concentration colony sweeps provides a work around but reduces the resolution of combinatorial expression profiles (termed phasotypes). A parsimonious approach combining both single colony and sweep data was developed to overcome these limitations. The critical methodological advance is the use of an algorithm that utilises the experimental data from the two sample types and a parsimonious, iterative mathematical analysis that outputs the phasotype distribution with the highest likelihood of underpinning the experimental data sets. The advantages of this unified method are increased resolution and accuracy of gene expression state combinations as compared to conventional single colony sampling, reduced requirement for sampling large numbers of colonies leading to reduced costs, and a higher capacity for collecting samples and replicates.

• Inputting of sweep and single colony data into an algorithm for a rapid determination of the combinatorial phase variation states (phasotypes) for repeat-mediated phase-variable bacterial genes
• This method reduces the number of single colony samples required to produce accurate estimates of phasotypes
• This method will reduce the costs of phasotype analyses and increase potential to analyse more time points or sample sites leading to an improved understanding of how phase variation contributes to bacterial host persistence and the ability to cause disease

## Graphical abstract

**Figure:**
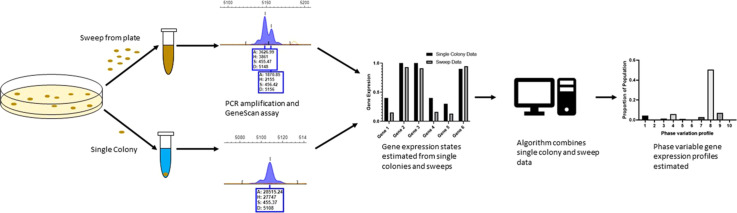

Specifications table

**Table:** 

Subject area:	Biochemistry, Genetics and Molecular Biology
More specific subject area:	*Phase variation*
Name of your method:	*Sweep-Corrected Phasotype Analysis*
Name and reference of original method:	Lango-Scholey L, Aidley J, Woodacre A, Jones MA, Bayliss CD (2016) High Throughput Method for Analysis of Repeat Number for 28 Phase Variable Loci of *Campylobacter jejuni* Strain NCTC11168. PLoS ONE 11(7): e0159634. https://doi.org/10.1371/journal.pone.0159634
Resource availability:	N.A.

## Background

Microbial population dynamics are subject to change due to environmental pressures and stochastic events that result in bottlenecks. A bottleneck refers to a rapid reduction in population size either due to selection, by the presence of specific environmental pressures that act against certain phenotypes, or non-selectively through random elimination of portions of the population regardless of phenotype [1], [2], [3]. Pathogen-host interactions have the potential to elicit both types of bottlenecks; for example, non-selective bottlenecking during spread within a host or transmission between hosts; and selective bottlenecking, for example, as a result of antigen-specific immune responses. Each of these events results in a reduction in the bacterial population. To counter these events, genetic and epigenetic mechanisms have evolved that generate significant levels of phenotypic heterogeneity and plasticity within bacterial populations and hence maintain fitness and the likelihood of species survival upon exposure to these bottlenecks. These mechanisms are localised to specific genomic regions, termed contingency loci, and result in microbial diversity being strongly linked to microbial survival for some bacterial pathogens and commensals.

Phase variation (PV) is mediated by heritable genetic mechanisms that generate intra-clonal diversity in asexually reproducing bacteria and is one outcome of localised hypermutation within contingency loci. One mechanism of PV is mediated by slip strand mispairing of hypermutable DNA regions containing simple sequence repeats (SSR). Expansion or contraction of the number of repeats in the SSR due to slip strand mispairing affects phenotypic expression of the contingency locus [4,5]. Changes in gene expression can result from the expansion or contraction of intragenic SSR, leading to introduction of premature stop codons into the reading frame. Conversely, alterations in intergenic SSR can impact promoter or enhancer sequences, ultimately affecting the binding affinity of regulatory factors. Several bacterial species contain multiple phase-variable genes whose combinatorial alterations are referred to as the phasotype of a bacterium. The number of potential phasotypes is affected by phasome size (i.e. the number of phase variable genes in the genome) and the number of expression states of each phase variable gene [6]. Finally, the stochastic nature of PV and high mutation rates allow for rapid generation of phenotypic heterogeneity within a clonal population and a concomitant increase in diversity after each generation.

Experimental analyses of PV are important for improving our understanding of microbial adaptability and determinants of microbial survival and replication of a number of important bacterial pathogens (e.g. *Neisseria meningitidis* and *Campylobacter jejuni*). Current methods for detecting repeat tract changes include, but are not limited to, whole genome or gene sequencing across single colonies or pooled isolates [5,7], ELISA or immunoblotting against single colonies [8,9], or through fragment length analysis against single colonies [10,11]. Advances in fragment length analysis, through GeneScan of multiplex PCRs and the development of automated tract length determination, have acted to reduce the workload on PV analysis pipelines [10]. However, logistical issues still remain such as isolating and analysing sufficient single colonies for accurate predictions of PV states while maintaining reasonable workloads. With longitudinal assays requiring numerous sampling steps even low single colony counts can scale rapidly. In order to achieve high quality phasotype estimates while reducing the number of single colonies required, we propose a hybrid approach that aims to simultaneously reduce the effect of random sampling bias, arising from the collection or availability of limited numbers of colony samples, and improve accuracy.

## Statement of the problem for predicting phasotypes from ‘sweep’ data

**Fig. 1:**
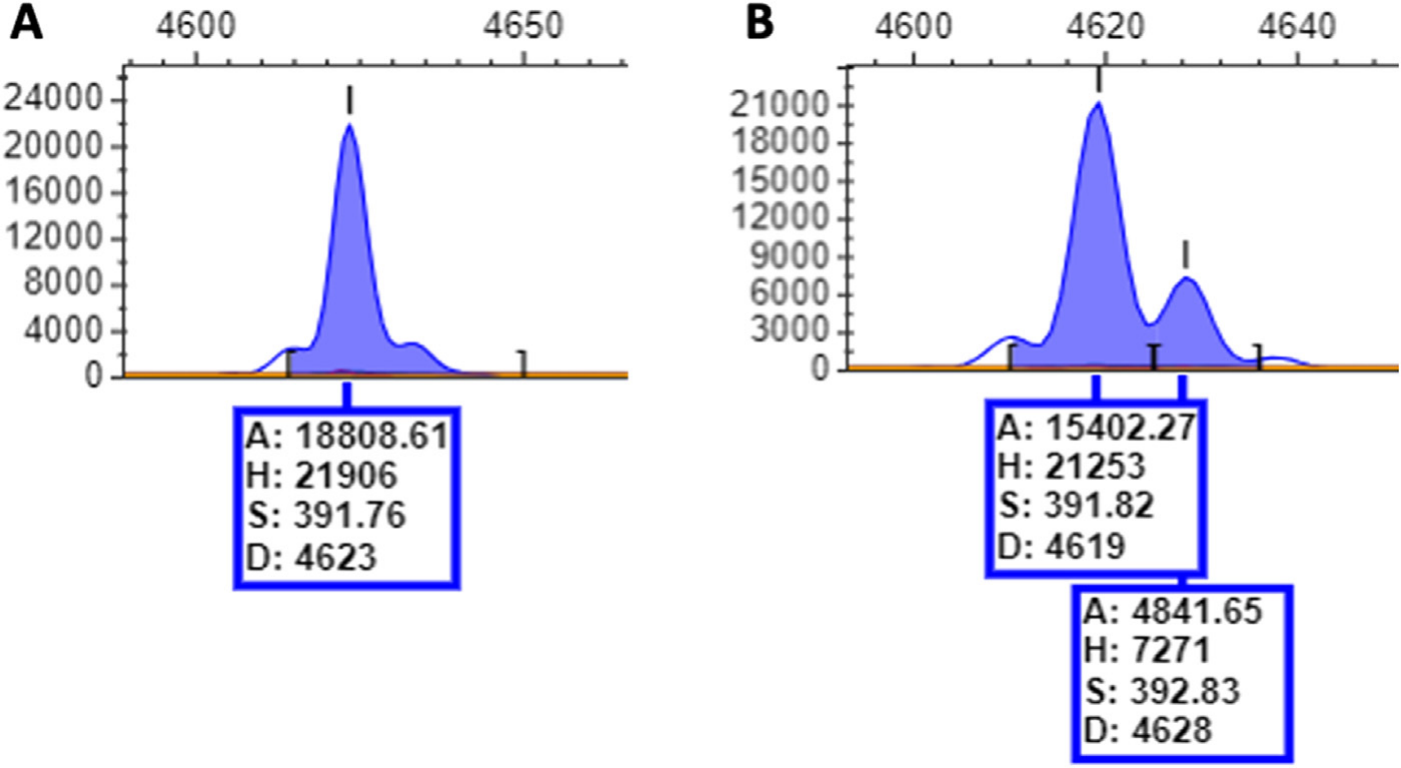
GeneScan of a single colony and a population sweep for a phase-variable gene. An example of the set of peaks obtained from a single colony (A) and a sweep (B) for a gene of length ∼392 base pairs as visualised in PeakScanner v2. The single colony (A) shows a single major peak of height 21,906 with no identified secondary peaks, while the sweep (B) shows an additional peak of ∼393 base pairs in length. The minor peak has a height of 7271 compared to the major peak of 21,253. *The metadata for each peak is as follows: A = area of peak, H = height of peak, S = size of peak in base pairs, D = datapoint*.

A ‘sweep’ refers to collection of a large number of multiple bacterial cells from a population. The sweep is derived from a low dilution plate of a serial dilution of a bacterial population. This sweep can be utilised to determine the major and minor expression states for each PV gene within the population. The analyses involves measuring the relative areas under the GeneScan peaks for different repeat numbers of an SSR tract (Fig. 1). These average expression states cannot, however, be utilised to determine the combinatorial expression states of the PV genes as different combinations of phasotypes can be produced by different single gene proportional states (see Fig. 1). For example, 25 % ON for gene A and 75 % ON for gene B could mean either 75 % 0–1 and 25 % 1–0 or 25 % 1–1 plus 50 % 0–1 and 25 % 0–0 (with ON and OFF coded as 1 and 0, respectively). Thus, the preferred method for derivation of phasotypes is analysis of single colonies. This method is however subject to the limitations of sampling (see Method Validation).

Single colony repeat numbers for a specific phase-variable gene are determined by PCR amplification of the repeat tract with fluorescently-tagged primers. These PCR products are subject to electrophoresis on an autosequencer (termed a GeneScan) and fragment length calling, relative to a size standard ladder, from output files with a software package such as PeakScanner. The most abundant PCR product produces a signature peak reflecting the PCR product length and is termed the major peak. Several smaller peaks, termed minor peaks, may surround the major peak but only the latter is considered for determining the repeat number of the colony. This fragment is then compared to a control PCR product of known repeat number and expression state.

Contrastingly, the sweep data set may contain a combination of multiple populations with varying fragment lengths due to differences in the repeat tract lengths. PCR amplification and GeneScan of this population produces a series of peaks whose heights/areas are proportional to the repeat numbers in the population. In order to estimate the expression state(s) for the sweep, all peaks are considered and sub-divided into repeat numbers that produce ON and OFF states. Following this division, the combined total for the peak heights (or areas) of the ON peaks is divided by the combined total for all peaks. For example, if the total peak height showing an ON state is 21,253 while the total peak height for the OFF state is 7,271, then the ON % for that sweep would be ∼75 % (i.e. 21,253/28,524; Fig. 1). Note that peak height and area are to a degree interchangeable but that PeakScanner software will sometimes overestimate areas as the edges of peaks are not always clearly defined (as can be seen in Fig. 1A).

## Method development

### In silico evidence for sweep correction improvements

To test whether sweep data can be utilised in combination with single colony analyses to accurately predict phasotype distributions, the algorithm was tested *in silico* with known distributions of the phasotypes. These analyses also set out to determine the lowest number of single colonies required for accurate predictions. These tests were run with a synthetic bacterial cell containing six phase-variable genes and hence capable of producing a maximum of 64 phasotypes (000000..101010…111111). To generate realistic populations, a simple switching algorithm was developed. For a population of 64 phasotypes, an individual phasotype was selected through weighted random selection acting on its proportion within the total population. The phasotype was then subject to a single gene switch (i.e. 000000 to 10000) and then re-added to the population. This produces a normal like distribution around a defined starting population. This process was repeated 20 times to produce 20 unique pseudo-populations with known proportions of each phasotype. The final phasotype distributions were utilised to calculate the ON % for each gene.

To mimic the single colony sampling method of experimental protocols, phasotypes were selected randomly from each of the 20 pseudo-populations using a random number generator and the proportion of each phasotype in the whole population. Multiple cumulative samples were generated that contained between 1 and 100 phasotypes. This method simulates picking single colonies from dilution plates of a large bacterial population with the number of samples being equivalent to the number of colonies. The next step was to utilise the algorithm to correct the single colony sampling data. The input data for the algorithm is the ON % for each gene, as calculated for the whole pseudo-population, and the proportions of each phasotype, as calculated from the 1 to 100 sample populations (see Fig S1 for an example run of the algorithm including outputs). This correction was performed for each of the sample sizes and all of the 20 pseudo-populations.

In order to assess the effectiveness of the algorithm in making improved predictions about the phasotype distribution of a sampled population compared to the real distribution, the Jensen-Shannon divergence equation was selected to determine the similarity of two distributions. A normalised distribution of phasotypes was used to seed each run of the algorithm. This distribution can be assumed to be a probability distribution, where each phasotype is represented as a proportion of the population. Two identical distributions of phasotypes (i.e. for 2 genes, we may have the four phasotypes, 11:01:10:00, present in the following proportions, 0.25:0.25:0.25:0.25, in both the sampled and real distributions) will produce a Jensen-Shannon divergence of 0 whereas a maximum value of 1 shows the highest possible divergence between the two distributions (e.g. phasotype proportions of 0.5:0.5:0:0 versus 0:0:0.5:0.5). Two scenarios were selected to test the robustness of the sweep corrected algorithm, a highly diverse input population where each phasotype is selected randomly (as outlined above), and a low diversity input population were a single phasotype is selected. This latter approach results in the selected phasotype forming an increased fraction of the population (i.e. 50 % of the population). These scenarios represents two possible situations often encountered during PV testing: A) a population under neutral selection; or B) one under positive selection.

**Fig. 2:**
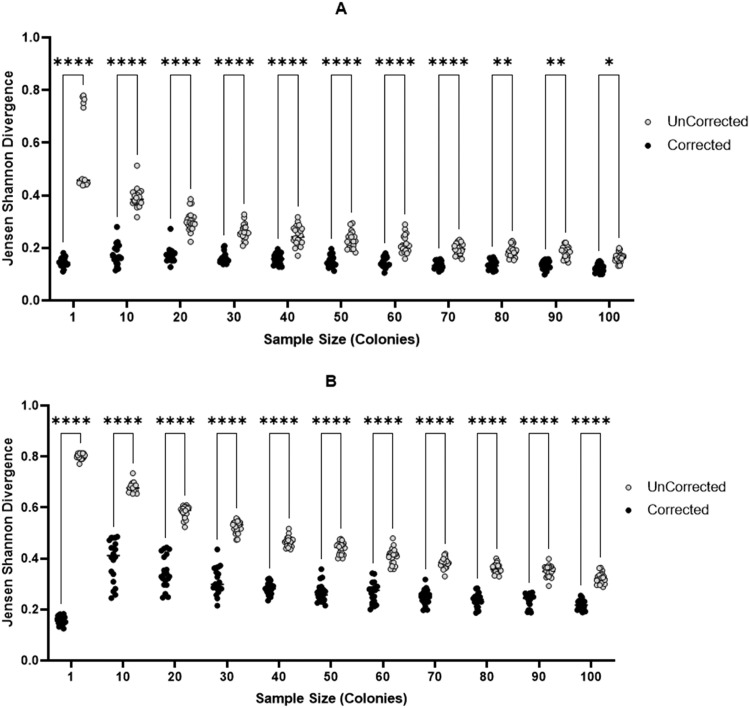
*In silico* comparison of single colony and sweep corrected methods for determining phasotype proportions in bacterial populations. Synthetic bacteria containing 6 phase-variable genes that are capable of generating 64 phasotypes were utilised to generate 20 populations of randomly selected phasotypes. Phasotype distributions for each population were determined by picking 1 to 100 samples (equivalent to a single colony in experimental protocols) and leaving this data uncorrected (single colony only) or applying an algorithm that corrects based on the ON % proportion for each gene (equivalent to ‘sweep corrected’ data in experimental protocols). The divergence of sweep corrected (black) and uncorrected single colony only (grey) phasotype distributions were calculated relative to the known input populations. These calculations were performed for a range of sample sizes using the Jensen Shannon Divergence equation. Differences in the distribution between the corrected and uncorrected data sets were tested for significance using an ANOVA test for variance for each sample size, correcting for multiple hypothesis testing (significance values ****: 0.0001,***: 0.001,**: 0.01,*: 0.05). Populations were sampled from either a clonal low diversity (A) or a high diversity mixed (B) population.

Across both *in silico* scenarios (Fig. 2), the sweep corrected datasets show lower divergence from the ‘true’ phasotype distribution at low single colony sample sizes (1–50) for the low diversity (A) as compared to the high diversity (B) populations. There is, however, a trend for both datasets to have similar levels of divergence by 100 colonies. The uncorrected single colony sampling method shows the same trend across both scenarios with a divergence of ∼0.8 (B) for most of the single colony samples but reducing to ∼0.2 (A) and ∼0.4 (B) with 100 samples. Comparatively, the sweep corrected data set shows less variability between the two scenarios, with divergence scores of 0.2 and 0.3, respectively, for most of the sample sizes. The clonal model (Fig. 2A) shows a closer match in the divergence scores between the sweep corrected and uncorrected data sets with a low area between the two curves for 50 or more samples. Thus, sweep correction has a reduced advantage for low diversity populations but is clearly advantageous for analysis of mixed populations. For the mixed population, a sample size of 10 single colonies with sweep correction produces a 57 % accuracy as compared to 100 single colonies in the uncorrected analysis (measured by the difference in average divergence; see Supplementary Data Accuracy Determination), the accuracy increases to 99 % when compared to 40 single colonies. Hence, sweep correction can significantly improve confidence in the prediction of the phasotypes when using small sample sizes.

Another way to consider the accuracy of these methods is to examine the proportions of each phasotype. When these proportions were examined for a sample size of 100, divergent patterns were observed for the two methods (Fig. S4). Thus the sweep corrected data has a tendency to overestimate some rarer phasotypes while underestimating the major phasotype. Conversely, the single colony sampling method overestimates the dominant phasotypes and in general underestimates the rare phasotypes but with occasions where one or a few rare phasotypes are overestimated due to chance picking of a rare phasotype. Overall, both methods provide a very close match to the true distribution at 100 samples with the over and underestimated values not producing a significant variation in phasotype proportion. Importantly, when sample size is reduced to 20 colonies, the single colony method produces significant over and under estimates of rare phasotypes (Fig. S5).

**Fig. 3:**
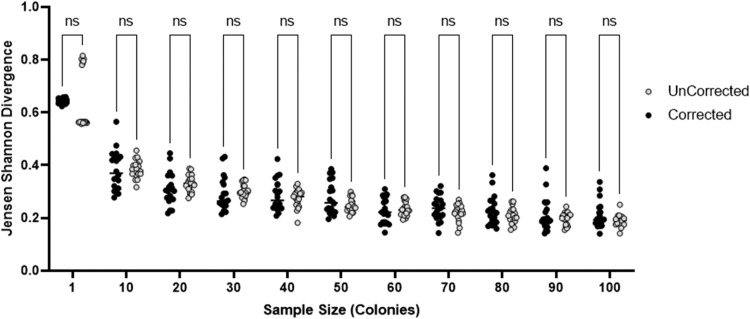
Assessment of the accuracy of the phasotype determination methods for a bimodal population. Comparison of the divergence scores for the sweep corrected (black) and uncorrected single colony only (white) sampling methods across a range of sample sizes for a bimodal population containing the 000000 and 111111 at similar, high (∼33 %) proportions. Each distribution between the corrected and uncorrected was tested for significance using an ANOVA test for variance for each sample size and correcting for multiple hypothesis testing (significance values ****: 0.0001,***: 0.001,**: 0.01,*: 0.05).

As a further test, a bimodal example was examined wherein two phasotypes, 000000 and 111111, were chosen to each form ∼33 % of the total population with the remaining phasotypes being randomly distributed by application of the switching method described previously. This scenario represents a rare situation where a population is split between two major divergent phasotypes. In this case, sweep correction only marginally outcompetes the single colony sampling method with sample sizes of >1 (Fig. 3). This figure also shows that ∼20 colony samples provide a reasonable estimate of the phasotype distributions in these populations.

In all cases, the Jensen Shannon divergence score did not reach 0 by 100 samples. This is in part due to the randomness of sampling. In order to achieve a divergence score of 0, for the *in silico* generated data, the sample size would need to identical to the total size of the population. More of less samples will always lead to some phasotypes being over-sampled. For example, if a population has 4 members across 4 phasotypes (00: 0.25, 10: 0.25, 01: 0.25, 11: 0.25), a divergence score of 0 can only be reached with samples sizes of 4, 8, 12, etc. and these samples must have identical proportions for each phasotype (i.e. 3 single colonies for each phasotype with a sample size of 12). A sample size of 30 (non-divisible by 4) cannot produce a divergence of 0. In the case of the *in silico* data in Figs. 2 and 3, the population size is 100 and therefore across 64 phasotypes the probability of a 0 divergence score is minimal. As a result, the correction will consistently yield a different but similar distribution. Supplementary data (Figure S3) shows a steady decline in divergence as sample size increases eventually reaching a close to 0 approximation (0.001) by 1 million samples.

### Considerations for the optimal sample size

Multiple SSR phase-variable genes are present in a diverse range of bacterial pathogens and commensals. Stochastic, independent switches in expression of these genes can give rise to high levels of variants, termed phasotypes, with combinatorial differences in expression of these genes. These phasotypes are potential determinants of important bacterial phenotypes and measures of the impact of population modulators such as bottlenecks. An accurate method for analysis of phasotype distributions is therefore critical for assessing changes in phase-variable populations during evolution both within and between hosts. Phasotype analyses have relied on sampling of small numbers of colonies with consequent sampling and resolution issues. The method outlined here describes a novel algorithmic approach to eliminating random selection bias or insufficient sampling and an overall improved resolution. Our tool combines both experimental sweep and single colony data to provide a more accurate picture of the phasotype distributions than currently achieved by the current approach of using low numbers of single colonies alone. The use of sweep data has been under utilised to date due to the risks associated with slippage in SSRs occurring during PCR reactions that create biases in the phasotype distributions. A PCR correction is applied to the sweep data prior to input into the sweep correction algorithm leading to a further enhancement in the accuracy of this approach.

In general, the *in-silico* modelling of various scenarios showed that sweep correction of the distribution of phasotypes leads to more accurate determination of the phasotype population distribution than colony samples alone (Fig. 2). These figures also show that 10 colonies produces a similar level of accuracy as compared to 30 colonies (note that 20 to 30 colonies have been utilized in most publications on phasotypes to date). The sweep correction is however minimal for bimodal populations due to skews in the sampling of the two major phasotypes, which the algorithm attempts to correct by making single gene changes rather than changing all genes simultaneously (as this would violate the parsimony approach). Overall, the parsimony approach used here is generally accurate for clonal populations subject to either genetic drift or selection. The weaker outcomes for bimodal distributions can be controlled for by checking to see if the single colonies and sweep data show a trend towards highly bimodal distributions.

## Methods details

### Description of the iterative algorithm for improving sampled phasotype distributions

Phase-variable genes are assumed to be able to switch between an ON (1) and OFF (0) state stochastically, reversibly and independently. As such, the probability of a single gene switch is significantly greater than the probability of 2 or more gene switches for a given phasotype.

The probability (p) of n gene switches occurring simultaneously for a total of N genes within a phasotype is:

$$p\left(n\right)=0.001,\;\;0.001^{2},\ldots ,\;\;0.001^{n}$$

For the simplest case of a single bacterium, and hence a single phasotype, this means that only a limited number of phasotypes are likely to be occupied by the majority of bacteria within the population. Additionally, the phasotype with the greatest number of gene switches, as compared to the starting phasotype, will comprise the lowest proportion of the population. This mutational pattern applies limitations to the potential mutational space occupied by a specific expression pattern for individual genes and hence provides a framework for predicting the phasotypes. This framework is the basis for the method elaborated herein. It should be noted that growth of a population through ‘enough’ generations, and in the absence of any selective or non-selective bottlenecks, will eventually lead to generation of a population containing all possible phasotypes. This latter occurrence is likely to be a rare observation in natural populations.

Our approach is to combine the minimum parsimony described above with ON % data, generated through a GeneScan analysis of sweep data, and phasotype distributions calculated from a small number of single colonies. This process involves application of a series of iterative steps starting with data from single colonies to seed the system and generation of a new phasotype distribution for each iteration of the algorithm. The phasotype data for N genes is normalised based on the total number of phasotypes within the distribution such that the total population will sum to 1. The input data are, therefore, the ON % for each PV gene in a phasotype derived from analyses of sweep data and the proportions of each phasotype derived from the single colony analysis.

### Method

The assumed starting point for this method is multiplex PCR and GeneScan data for multiple phase-variable genes as performed on a sweep and multiple single colonies derived from a specific bacterial population. The sweep and single colonies should be obtained from the same serial dilution of this bacterial population. The sweep should be obtained from a plate where there is either confluent growth or 1000+ colonies and the single colonies from plates where there are isolated single colonies. To compute the sweep adjusted phasotypes for this population, the following information must be provided, the number of genes, the ON % for each gene as determined from the sweep data (see Step 1) and the phasotypes of each single colony (Step 2). An example of the input is provided and outlined in Fig S1.

Step 1 **Determine ON % from sweep data**

1. Import the autosequencer file for the sweep data into the PeakScanner software (or other appropriate software) and analyse with the relevant size standard for the samples (e.g. GS600LIZ).
2. Examine the peaks for the sample and identify the major and minor peaks for each repeat tract.
3. Output the areas for the major and minor peaks.
4. Assign repeat numbers to major and minor peaks relative to a control of known repeat number.
5. Assign ON and OFF states to peaks based on known associations between the repeat number and in frame translation of the gene sequence.

Step 2 **Analyse single colony data for proportions of slippage**

1. Analyse the GeneScan data for multiple single colonies as described in Step 1.
2. For each gene, determine the areas of all major and minor peaks.
3. Calculate the total area for all the combined minor peaks and divide by the total area of all peaks to obtain the % area for the minor peaks.
4. For each gene, determine the % area of the minor peaks across 10 or more single colonies for samples where the major peak has the same number of repeats.

Step 3 **Correct ON % for slippage**

1. Calculate the average % area for the minor peaks from multiple single colonies (see Step 2) as a measure of the degree of slippage occurring during the PCR reactions (note that the correction is applied utilizing peaks of the same repeat number and for the same gene).
2. Subtract the % area for the minor peaks obtained from single colonies from the % area for the minor peaks in the sweep data.
3. Add the % area for the minor peaks obtained from single colonies to the area for the major peaks in the sweep data.
4. The output data has now been subject to sweep correction and can be utilized as input for Step 4.

Step 4 **Input data into the algorithm**

The algorithm is run in Python v3.9.5 and a suitable computer is required for utilizing this program. The program can produce data for phasotypes of 2 or more genes. The expression states of each gene are coded as 1 for ON and 0 for OFF.

1. Open program.
2. Input phasotypes of single colonies.
3. Input ON % values for each gene as derived from sweep data.
4. Indicate number of iterations.
5. Run algorithm.

Step 5 **Run algorithm**

Each iteration of the algorithm goes through the following steps:-

1. The distribution of phasotypes derived from the experimental single colony analyses is converted into a series of ON % for each gene in the phasotype.
2. Divergence between the values for ON % for each gene in the system derived from the sweep data versus the single colony phasotype distribution is calculated and the largest variance in ON % between the sweep and colony is used to identify the gene requiring the largest correction.
3. All phasotypes present in the distribution are considered for potential changes. This process leads to identification and selection of those phasotypes whose ON or OFF states could be switched to reduce the divergence in the ON % of the identified gene.
4. The percent change required to align the ON % for a specific gene from the phasotype data to the sweep data is proportionally distributed between all selected phasotypes. This process simultaneously decreases the relative representation of the most divergent phasotypes in the population while increasing those phasotypes most closely aligned with the observed expression state.
5. This new population then becomes the new phasotype distribution. The algorithm goes through multiple cycles adjusting each gene individually until either there is convergence - where the difference in gene ON % is the same between the new phasotype distribution and sweep data - or a user defined number of cycles is completed.
6. The final output file contains the corrected gene ON % for the population and estimates of the proportions for each possible phasotype within the population.

For each cycle of the algorithm only a single PV gene state is considered for each phasotype in the system, therefore producing the minimum number of state changes required to mirror the percentage of gene ON % between the phasotype and sweep data.

Step 6 **Perform check of distributions of the phasotype output of the algorithm**

1. Utilise the single colony data to derive the phasotypes (see Step 4 for coding of gene expression states).
2. Determine the phasotype for the required combination of genes for each single colony.
3. Determine the numbers of each phasotype across all analysed single colonies.
4. Calculate the percentage of each phasotype.
5. Compare the single colony phasotype data to the sweep corrected data and examine for evidence of major discrepancies (e.g. absence or major reduction in the frequency of a phasotype in the sweep corrected data).
6. Where such differences are observed, examine the single colony data for evidence of a bimodal distribution of two phasotypes that have non-overlapping combinations of expression states.
7. Flag these samples as potentially requiring further investigation (e.g. sampling of more single colonies) or low confidence.

## Method validation

### In vitro confirmation of hybrid approach to predicting phasotype distributions

Step 1. **Serial Passage**

*C. jejuni* strain NCTC 11168ca was grown for 24 h in Brucella broth (6 mL). After this initial incubation, a sample of 600 µL was transferred to a new liquid culture (6 mL: i.e. a 1 in 10 dilution) and regrown for another 24 h. This cycle was repeated across five days in order to increase diversity within the bacterial population. After the last cycle, a serial 10-fold dilution was performed in triplicate with Brucella broth as diluent. A 10 uL aliquot from each dilution was spread with a sterile cotton swab onto a blood-agar plate and incubated for 2–3 days at 42 °C under microaerophilic conditions:- 83 % N_2_, 10 % CO_2_ and 7 % O_2_ in a VAIN cabinet (Variable Atmosphere Incubator; Don Whitley Scientific Ltd, Shipley, UK). Multiple single colonies (*n* = 287) were picked and individually re-suspended in deionised water in non-skirted PCR plates. Bacterial sweeps were taken from 10°, 10^1^ and 10^2^ dilution plates, re-suspended in deionised water and aliquoted into non-skirted PCR plates. The single colonies and sweep samples were then denatured by heating to 94 °C for 5 min in a PCR machine.

Step 2: **GeneScan of multiple phase variable genes**

A total of 6 phase-variable genes were selected and the repeat tracts of these genes were amplified from all single colony and sweep samples using multiplex PCR reactions, as described by Lango-Scholey et al. [10]. One PCR primer of each primer pair was labelled with a fluorescent FAM tag [10]. Amplification from a set of single colony PCRs was confirmed by electrophoresis of 5 uL aliquots of each reaction on a 2 % TAE agarose gel. Table 1 shows the target locus and PCR product size. Additionally, a set of control samples were amplified from a genomic DNA sample that had been subject to sequencing of the repeat tracts (Cayrou and Bayliss, unpublished data).

**Table 1:** Phase variable gene selected for multiplex reaction.

Locus	PCR Product Size (bp)	PCR product repeat number	In-frame repeat number
*capA*	457	10G	11G
*cj0031*	221	10G	9G
*cj0045*	280	10G	11G
*cj0685*	128	9G	10G
*cj1325*	165	10G	9G
*cj1342*	392	9G	9G

Following confirmation of the expected PCR products, an A-tailing reaction was performed on the PCR products as previously described [10]. Samples were then either stored at −20 °C or taken directly for GeneScan. The GeneScan analysis was performed using a mixture of 1 µL of A tailed multiplex PCR product, 0.5 µl of GS600LIZ size standard ladder (ABI) and 9.5µL of deionized formamide. GeneScan analyses were carried out on an ABI3500 series genetic analyser through capillary electrophoresis. PCR product sizing was carried out using the ThermoFisher cloud dashboard, selecting a size standard of GS600LIZ, to generate fragment lengths for each of the six genes in the multiplex. The complete set of peak sizes were extracted and gene ON/OFF states were estimated for each single colony using PSAnalyse and reference files with the known repeat numbers and expression states for the reference genomic DNA [10].

The ‘sweep’ GeneScan analyses were utilised for determining the relative proportions of repeat numbers that correlate with the ON and OFF states by determining the areas under the major and minor peaks observed in the ThermoFisher dashboard. The ON % was determined by dividing the area of the peak representing the ON number of repeats by the area of all combined peaks and multiplying by 100. Finally, a correction for PCR slippage was applied to the sweep gene ON percentage. Slippage in the repeat tracts occurs during the PCR reactions and can alter the estimated size of the major and minor peaks. The slippage correction was calculated utilising the PCR data from the single colony GeneScan data as the majority of single colonies will contain a low number of variants due to derivation from a single cell and to the low number of generations. For each colony, the ratio of the area of the minor to the major peak was selected from the data in the PSAnalyse output file, and an estimate of the average level of slippage across multiple colonies was generated for each gene. A value for slippage rate (as a % from the main peak) was estimated by taking the average major to minor peak ratio and dividing it across the total number of single colonies to gain an average combined −1 and +1 repeat peak error rate per colony for each gene (ranging from 0.3 to 17 %). This was then applied to the sweep data by correcting the values for the major and minor peaks to give a new ON %. For example, if all single colonies for a given gene show a secondary peak of 10 % of the height or area of the major peak, then this is an indication that slippage has occurred within the single colony PCR reactions and will also have occurred in the sweep PCR reactions. Therefore, before estimating the ON % for a given sweep set, 10 % of the major peak data should be subtracted from the minor peak data and added to the major peak data (e.g. if the major peak has a height of 7000 and the minor peak is 2000; a 10 % rate slippage from major to minor (700) peak means that the true values for these major and minor peaks are 7700 and 1300, respectively).

### Evaulation of sweep correction for experimental data sets

**Fig. 4:**
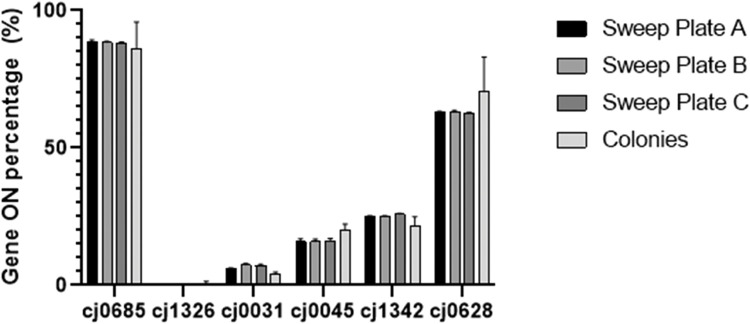
Comparison of the ON % ratios of 6 phase variable genes from sweep and single colony experimental data sets. ON status across 9 (three dilution series in triplicate) collected sweeps and 287 colonies sampled from the three populations. *The mean ON % is represented by bar height with the standard error from mean (SEM). The Sweep data set was corrected for slippage prior to plotting*.

Experimental data sets of phasotype distributions were generated by determining the PV states of six genes for multiple single colony isolates from low dilution plates and sweeps from a high dilution plate of the same *C. jejuni* population in triplicate. The ON % states for six genes were calculated from either the phasotypes of the 288 single colony analyses or from the sweep peak ratios. Three sweep duplicates were combined for each sweep data set while all colonies were combined to build the gene ON % values. The values for the ON % for each of the colony datapoints were corrected for slippage against the single colony values (this corrected a major difference for *cj0031*, see Fig. S2 in comparison to Fig. 4). This is an important correction as slippage during the PCR can artifically inflate the areas of the minor peaks that are being used to estimate proportion of the minor PV state in the actual population.

The experimental data consisted of two genes with very high or very low ON % rates, *cj0685* and c*j1326* respectively, while other genes spanned a range between these extremes. For each of the six genes, the sweep triplicates have consistent ON % values across all 6 genes, with the mean of each triplicate set varying by no more than 5 %. In comparison, the colony mean ON % values are in close agreement with the sweep data.

**Fig. 5:**
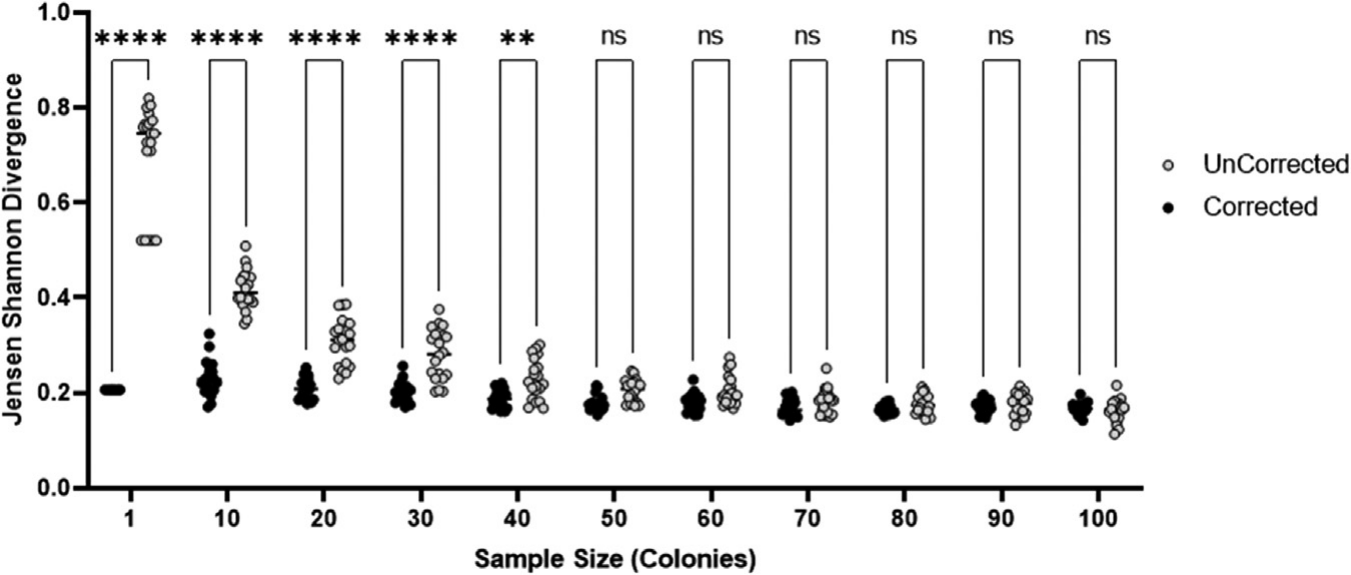
Application of the sweep correction method to analysis of an experimental phasotype distribution. The six gene phasotype distribution of a *C. jejuni* population was determined from a PV analysis of 288 colonies. Sweeps from high dilution plates of the same population were also subject to a PV analysis. The divergence from this ‘true’ population data was determined for phasotype distributions from 1 to 100 randomly sampled single colonies (grey circles) and after correction of these samples with the experimental sweep data (black circles). The differences in the distributions of the corrected and uncorrected data sets were tested for significance using an ANOVA test for variance for each sample size, correcting for multiple hypothesis testing (significance bounders ****: 0.0001,***: 0.001,**: 0.01,*: 0.05.

Due to the low variance in ON % ratios for the sweep data, an average ON % value for the three sweep data sets was utilised as input into the sweep correction model. The other input was the phasotypes for each colony, which was extracted from the analysis of the 287 isolates and used to define the distribution of each phasotype within the population. All phasotypes across all colonies were considered and randomly sampled based on their appearence in the population. A range of 1 to 100 samples were taken representing experimentally the number of single colony picks. Each single colony pick count was repeated 20 times, with the Jensen Shannon Divergence index calculated between the population of phasotypes for the ‘colony picks’ versus the known population based on the 287 colonies in the experiment. Each sample size was then corrected in parallel using the sweep gene ON %. The divergence from the true population is shown in Fig. 5.

The sweep corrected sampling method shows lower divergence from the true population compared to the uncorrected sampling population for all samples sizes of 1 colony to 40 colonies. At low sample sizes (i.e. <20 colonies), sweep corrected sampling showed a large reduction in the divergence score with the sweep corrected divergence score for a sample size of 10 colonies being 2-fold lower than for the uncorrected score. Across the corrected sampled populations the divergence scores are similar across all samples sizes ranging from a peak of 0.324 with 10 colonies to the lowest value of 0.14 with 100 colonies whereas the scores for the uncorrected samples range from 0.81 to 0.11.

**Fig. 6:**
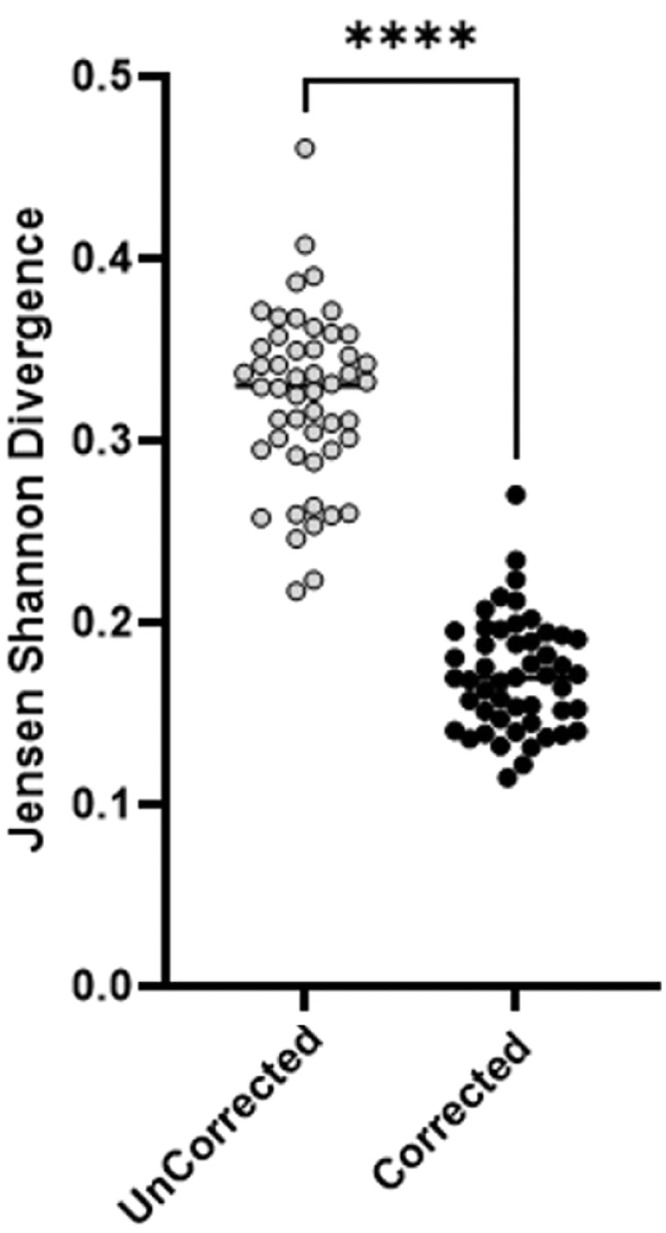
Comparison of sweep corrected and uncorrected 20 colony phasotype distributions. Divergence scores of phasotype distributions were calculated using a sample size of 20 colonies either with sweep correction (black circles) or uncorrected (white circles). A total of 20 colonies were sampled across 50 replicates. The scores for the two methods were compared using a Welch's T Test.

Commonly a range of between 18 and 30 [6] colonies are sampled to represent phasotype populations. To assess the validity of our corrective algorithm at these sample sizes a set of 50 random populations of 20 sampled colonies were generated from the in vitro data set and compared for divergence from the true phasotype distribution using the Jensen Shannon Divergence index. A Welch's T Test was preformed to compare the divergence scores generated from the two populaitons (Fig. 6).

The mean divergence score for the uncorrected population was approximately 0.3 as compared to 0.2 for the corrected population. The Welch's T Test returned a P value of <0.0035 indicating a significant change in the mean for the population from the sweep corrected distribution as compared to the uncorrected population for a sample size of 20.

### Evaluation of systemic errors in the sweep pcr data on the sweep correction method

**Fig. 7:**
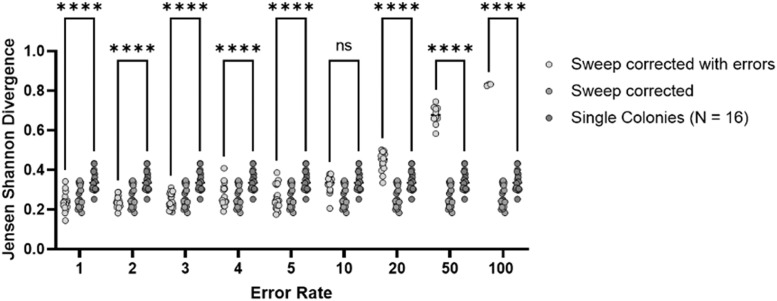
Assessment of the effects of PCR error rates on the accuracy of the sweep correction method. The six gene phasotype distribution as described in Figs. 4 and 5 was subject to application of a PCR error rate prior to calculation of the sweep correction data. Three scenarios were examined: single colonies only (dark grey), sweep corrected single colonies, sweep corrected single colonies with a randomised PCR error rate. 16 single colonies were sampled for the single colonies only with 7 selected for the sweep correction. An artificial error rate was introduced by randomly adding a factor (error rate) to the derived gene ON % from the sweep samples prior to correction. The divergence from the true phasotype distribution was measured using the Jensen Shannon divergence. The differences in the distributions across the single colony and error induced data sets were tested for significance using an ANOVA test for variance for each sample size, correcting for multiple hypothesis testing (significance bounders ****: 0.0001,***: 0.001,**: 0.01,*: 0.05.

All the comparisons described above were predicated on the assumption that ON % values derived from sweeps had a high accuracy relative to the actual population. However, the sweep PCRs may have inaccuracies due to the quality of the PCR, difficulties in determining slippage rates, peak height/size cut-off effects in the PeakScanner software or a bias in the sweep sampling. These effects may result in major differences between the sweep and actual populations in gene ON % values. The role of these potential error rates on estimating population phasotype proportions was assessed by simulating generic error rates. Twenty sets of 16 single colonies were randomly sampled from the in vivo data set. These samples were compared to the known phasotype distribution using the Jensen Shannon divergence. An additional 7 single colonies were randomly selected across 20 replicates and corrected using the sweep derived ON % states. These samples were also compared to the known phasotype distribution. Finally, the gene ON % values, as derived from the sweep data, were randomly altered by a fixed percentage error change (i.e. a 5 % error resulted in alteration of the ON % for a gene at 50 % to new values of either 45 % or 55 %) that ranged from 1 to 100 % (Fig. 7). For error rates of 10 % or lower, the sweep correction method still outcompeted the single colonies alone approach. For error rates of 20 %, 50 % and 100 %, the single colonies showed a significantly lower divergence from the true phasotype distribution. This effect highlights the importance of obtaining accurate ON % data from the sweep populations and also in comparing data obtained from single colonies to sweep corrected data as a sanity check to confirm that the correction method has not generated an aberrant data set.

### Assessment of samples size from method validation data

Both the new model and the current single colony sampling strategies were assessed for accuracy through a PV analysis of experimental data of 6 phase-variable genes. By combining known sweep data for a set of genes with a large volume (*n* = 288) of single colonies and correcting for PCR slippage using single colony data, we have shown that the ON % of a gene can be estimated accurately for a population through taking sweeps as shown in Fig. 5. The relative ON % states for each gene are shown to be highly conserved across several different sweep sets as compared to single colony data with only marginal differences in gene ON proportions. This comparison provides evidence that slippage-corrected sweep data is not only consistent across replicates but consistent between itself and combined single colony data and hence is a valuable standalone tool in predicting gene switching across phase-variable loci. The applicability of sweep mediated gene ON state estimation for the whole population also indicates that experimental sweep data is an appropriate input for the algorithm.

The application of the algorithm across a range of sample sizes was compared to uncorrected distributions. As shown in Figs. 4 and 5, the applicability of the sweep-single colony hybrid approach provides an improved sampling method. With a standard sampling size of 20 single colonies, the corrected population showed significantly lower divergence from the true population than the uncorrected population with a 50 % reduction in overall divergence. Thus, current sampling methods can be significantly improved by the adoption of our sweep correction method. Furthermore, the new method extends to low sample sizes, indicating that at very low sample sizes, such as 10 single colonies or less, our hybrid approach is able to outcompete standard single colony sampling of size 20–50. If we consider Fig. 5, the divergence with 50 samples is similar between the corrected and uncorrected samples (average 0.18 – 0.2). If we now compare this data to the average divergence value of 0.23 obtained for the 10 sample sweep-corrected data (note that there is a variance of 13 % for these samples), an accuracy estimate of ∼87 % (see Supplementary File Accuracy Determination for the method) is obtained for the sample of 10 colonies as compared to that of 60 single colonies without correction. We therefore recommend that when using sweep corrected data, a sample size of 10 is adequate for a resolution equivalent to 50 – 100 colonies in uncorrected samples and has a much higher accuracy than 20 or 30 colonies that has been utilized in most publications to data. Collection of a sweep and 10 single colonies will reduce the overall load of sampling and downstream analyses (e.g. PCR and GeneScan reactions) by 50 % or more and hence enable analysis of more time points and replicates. These modifications could result in major advances in experimental analyses of SSR-mediated phase-variable populations.

## Ethics statements

No ethical issues identified

## CRediT authorship contribution statement

**Jonathan Holmes:** Conceptualization, Methodology, Software, Validation, Formal analysis, Investigation, Data curation, Writing – original draft, Visualization. **Lickson Munjoma:** Validation, Investigation, Data curation, Writing – original draft. **Christopher D. Bayliss:** Conceptualization, Writing – original draft, Writing – review & editing, Supervision.

## Declaration of Competing Interest

The authors declare that they have no known competing financial interests or personal relationships that could have appeared to influence the work reported in this paper.

## Data Availability

Data will be made available on request.

## Appendix Supplementary materials

Image, application 1

Image, application 2

Image, application 3

Image, application 4

Image, application 5

Image, application 6

Image, application 7

Image, application 8
