# Supplementary material for: Novel method for prediction of combinatorial phase-variable gene expression states
Source: MethodsX. 2023 Sep 22;11:102392. doi: 10.1016/j.mex.2023.102392 (PMC10561117; doi:10.1016/j.mex.2023.102392)
Supplement: Supplementary file 7 [file mmc7.docx]

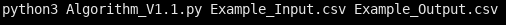

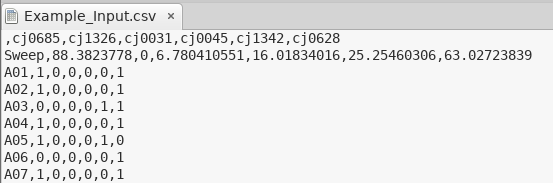

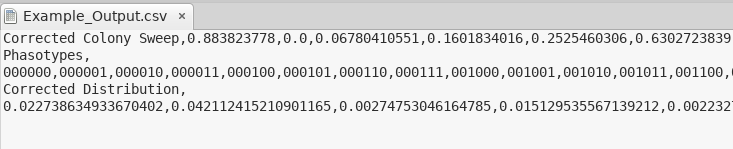


**S1. An example run of the algorithm on the *in vitro* dataset showing the expected input file the calling of the algorithm in python and the format of the output file.** The input file contains 3 sections the first line act as a placeholder containing the gene names, the second line holds the sweep data as a % ON expression, finally all subsequent lines hold the single colony data in the format well name followed by the ON or OFF state of each of the genes in that colony. The Algorithm is then called through the Terminal with the name of the python executable followed by the Input file and then the desired output file name. The Output file contains the Corrected Sweep data which will match the sweep data if convergence has been achieved in the number of iterations desired. The total possible phasotypes which could be present in the population. Finally, the corrected distribution of each phasotpye in the population as a fraction of the total population.


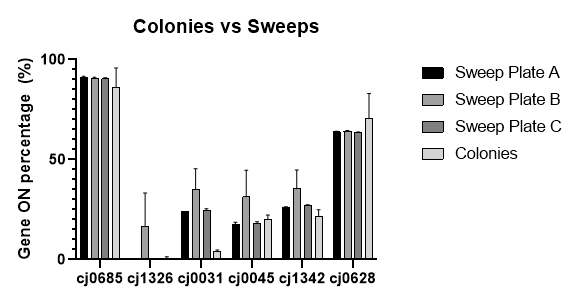


**S2. Comparison of the uncorrected ON % ratios of 6 phase variable genes from sweep and single colony experimental data sets.** ON status across collected 9 sweeps and 287 colonies sampled from the 3 populations. *The mean ON % is represented by bar height with the standard error from mean (SEM).*


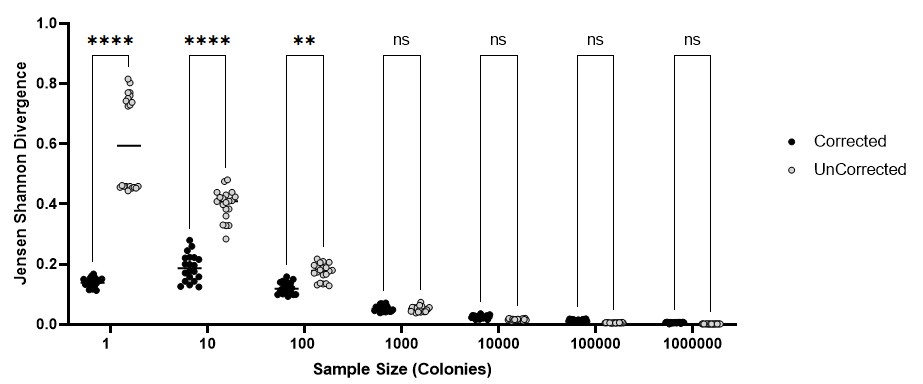


**S3. Assessment of the accuracy of the phasotype determination in a clonal populaiton beyond 200 samples.** A comparison of sweep corrected (black) to uncorrected sampling (grey) for an *in silico* population generated from a clonal population. As the number of samples increases the divergence continues to reduce as the number of samples increases. However a divergence of 0 is never reached.


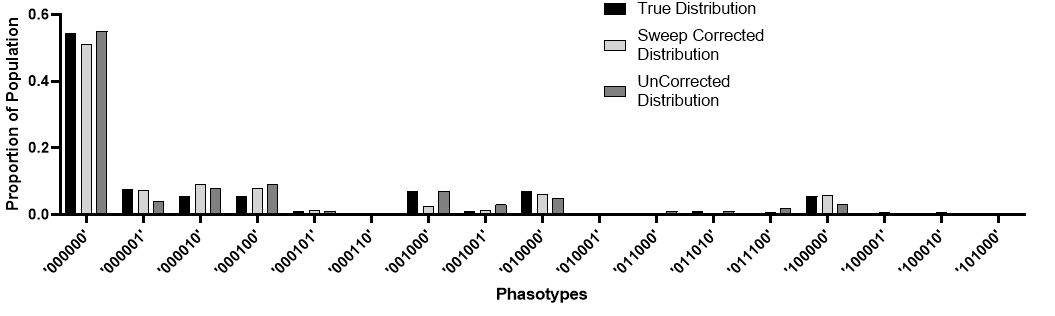


**S4. A comparison of the major phasotypes in a 200 sample example generated from an *in silico* model of a clonal population.** Each phasotype and its proportion is represent for those than contain more tha 1% of the true distribution to avoid over saturation of low value phasotypes. The true distribution (black), sweep corrected (light grey) and uncorrected (dark grey) proportions of each population is shown. The sweep corrected data shows a better fit on rarer phasotypes than sampling alone such as in 100000, 000001, 010000 an 000100 however appeast to underestimate some major groups such as 000000 and 001000.

**
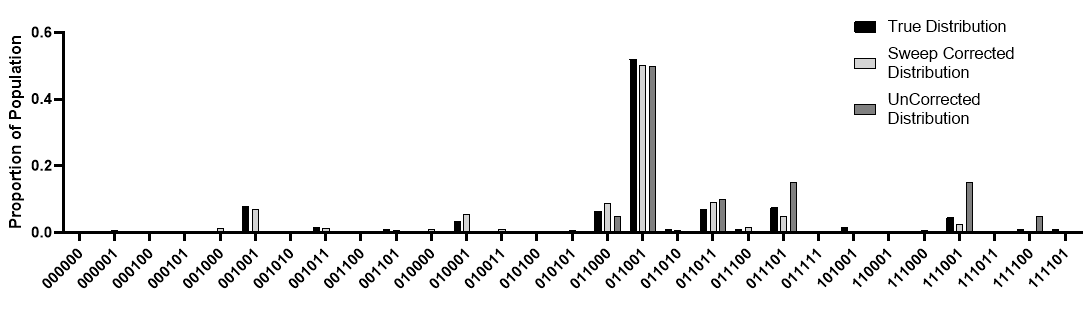
**

**S5. A comparison of the major phasotypes in a 10 sample example generated from an *in silico* model of a clonal population.** Each phasotype and its proportion is represent for those than contain more tha 1% of the true distribution to avoid over saturation of low value phasotypes. The true distribution (black), sweep corrected (light grey) and uncorrected (dark grey) proportions of each population is shown. The sweep corrected data shows a better fit on all phasotypes present in the population compared to the uncorrected distribution, while the sweep corrected may give under-represent some phasotypes sweep correcting accuractly estimated phasotypes 011011, 111001 and 001001 compared to the uncorrected distribution which was unable to accuratly represent this data and tends to overestimate some phasotypes while underestaimting others.
