# Supplementary material for: Novel method for prediction of combinatorial phase-variable gene expression states
Source: MethodsX. 2023 Sep 22;11:102392. doi: 10.1016/j.mex.2023.102392 (PMC10561117; doi:10.1016/j.mex.2023.102392)
Supplement: Supplementary file 8 [file mmc8.docx]

Supplementary Information. Determination of the Accuracy of Sweep Corrected Phase Variation Data.

Accuracy was measured using a standard equation of:

Accuracy =100−[(True value−Observed)/True value∗100]

For the data in Figure 2A, the following detailed approach was followed. The intention was to compare the accuracy of the sweep corrected data to uncorrected data obtained only from analysis of colonies. This was achieved by comparing the divergence between data obtained for 10 sweep corrected samples to 100 uncorrected samples. The sweep corrected data was generated by randomly selecting 10 colonies and correcting with the sweep data measurements using the method outlined in the text. The uncorrected data was generated from 100 random selections of 1 to 100 colonies.

The accuracy was determined by comparing: 1) the lowest divergence of the 10 corrected samples to the average divergence of 100 uncorrected samples; 2) the average divergence for the 10 sweep corrected samples compared to the 100 uncorrected samples.

The table outlines the accuracy values obtained from this data. Note that the sweep corrected 10 colonies is 99% as accurate as taking 40 single colonies on average

and at best the 10 sweep corrected colonies can be 94% as accurate as 100 colonies

|  | Accuracy compared to 10 sweep corrected samples | |
| --- | --- | --- |
| Number of colonies | Average | Best |
| 1 | 167.1388 | 175.6541368 |
| 10 | 144.7804 | 159.0895136 |
| 20 | 125.4325 | 144.7552315 |
| 30 | 117.8859 | 139.1641328 |
| 40 | 99.44378 | 125.5009555 |
| 50 | 87.43597 | 116.6047379 |
| 60 | 87.18193 | 116.4165254 |
| 70 | 74.98917 | 107.383281 |
| 80 | 69.55334 | 103.3560387 |
| 90 | 67.82709 | 102.0771115 |
| 100 | 57.89176 | 94.71633053 |
